# EEG complexity as a biomarker for autism spectrum disorder risk

**DOI:** 10.1186/1741-7015-9-18

**Published:** 2011-02-22

**Authors:** William Bosl, Adrienne Tierney, Helen Tager-Flusberg, Charles Nelson

**Affiliations:** 1 Harvard Medical School, Boston, MA, USA; 2 Children's Hospital Boston Informatics Program at Harvard-MIT Division of Health Sciences and Technology, Boston, MA, USA; 3 Harvard Graduate School of Education, Cambridge, MA, USA; 4 Department of Developmental Medicine, Children's Hospital Boston, Boston, MA, USA; 5 Department of Psychology, Boston University, Boston, MA, USA

## Abstract

**Background:**

Complex neurodevelopmental disorders may be characterized by subtle brain function signatures early in life before behavioral symptoms are apparent. Such endophenotypes may be measurable biomarkers for later cognitive impairments. The nonlinear complexity of electroencephalography (EEG) signals is believed to contain information about the architecture of the neural networks in the brain on many scales. Early detection of abnormalities in EEG signals may be an early biomarker for developmental cognitive disorders. The goal of this paper is to demonstrate that the modified multiscale entropy (mMSE) computed on the basis of resting state EEG data can be used as a biomarker of normal brain development and distinguish typically developing children from a group of infants at high risk for autism spectrum disorder (ASD), defined on the basis of an older sibling with ASD.

**Methods:**

Using mMSE as a feature vector, a multiclass support vector machine algorithm was used to classify typically developing and high-risk groups. Classification was computed separately within each age group from 6 to 24 months.

**Results:**

Multiscale entropy appears to go through a different developmental trajectory in infants at high risk for autism (HRA) than it does in typically developing controls. Differences appear to be greatest at ages 9 to 12 months. Using several machine learning algorithms with mMSE as a feature vector, infants were classified with over 80% accuracy into control and HRA groups at age 9 months. Classification accuracy for boys was close to 100% at age 9 months and remains high (70% to 90%) at ages 12 and 18 months. For girls, classification accuracy was highest at age 6 months, but declines thereafter.

**Conclusions:**

This proof-of-principle study suggests that mMSE computed from resting state EEG signals may be a useful biomarker for early detection of risk for ASD and abnormalities in cognitive development in infants. To our knowledge, this is the first demonstration of an information theoretic analysis of EEG data for biomarkers in infants at risk for a complex neurodevelopmental disorder.

## Background

The human brain exhibits a remarkable network organization. Although sparsely connected, each neuron is within a few synaptic connections of any other neuron [1]. This remarkable connectivity is achieved by a kind of hierarchical organization that is not fully understood in the brain, but is ubiquitous in nature and is called a scale-free network [2-4] that changes with development. Complex networks are characterized by dense local connectivity and sparser long-range connectivity [2] that are fractal or self-similar at all scales. Modules or clusters can be identified on multiple scales. A comparison of network properties using functional magnetic resonance imaging showed that children and young adults' brains had similar "small-world" or scale-free organization at the global level, but differed significantly in hierarchical organization and interregional connectivity [5]. White matter fiber tracking has revealed that brain development in children involves changes in both short-range and long-range wiring, with synaptogenesis and pruning occurring at both the local (neuronal) level and the systems level [5]. Abnormal network connectivity may be a key to understanding developmental disabilities.

Autism is a complex and heterogeneous developmental disorder that affects the developmental trajectory in several key behavioral domains, including social, cognitive and language abilities. The underlying brain dysfunction that results in the behavioral characteristics is not well understood. Complex mental disorders such as autism cannot easily be described as being associated with underconnectivity or overconnectivity, but may involve some form of abnormal connectivity that varies between different brain regions [6]. Normal and abnormal connectivity may also change during development, so that, for example, a condition may not exist at age 3 months but may emerge by age 24 months. A key to understanding neurodevelopmental disorders is the relationship between functional brain connectivity and cognitive development [7]. Measuring functional brain development is difficult both because the brain is a complex, hierarchical system and because few methods are available for noninvasive measurements of brain function in infants. New nonlinear methods for analyzing brain electrical activity measured using scalp electrodes may enable differences in infant brain connectivity to be detected. For example, coarse-grained entropy synchronization between electroencephalography (EEG) electrodes revealed that synchronization was significantly lower in children with autism than in a group of typically developing children [8], supporting the theory that autistic brains exhibit low functional connectivity. In the autistic brain, high local connectivity and low long-range connectivity may develop concurrently because of problems with synapse pruning or formation [9,10]. Estimation of changes in neural connectivity might be an effective diagnostic marker for atypical connectivity development.

EEG signals are believed to derive from pyramidal cells aligned in parallel in the cerebral cortex and the hippocampus [11], which act as many interacting nonlinear oscillators [12]. As a consequence of the scale-free network organization of neurons, EEG signals carry nonlinear, complex system information reflecting the underlying network topology, including transient synchronization between frequencies, short- and long-range correlations and cross-modulation of amplitudes and frequencies [13]. The mathematical relationship between network structure and time series is a subject of current research and may eventually shed further light on the relationship between neural networks and EEG signals.

A great deal of information about interrelationships in the nervous system likely remains undiscovered because the linear analysis techniques currently in use fail even to detect them [14]. If brain function and behavior are mirrors of each other as is commonly accepted [15-18], then biomarkers of complex developmental disorders may be hidden in complex, nonlinear patterns of EEG data. The dynamics of the brain are inherently nonlinear, exhibiting emergent dynamics such as chaotic and transiently synchronized behavior that may be central to understanding the mind-brain relationship [19] or the "dynamic core" [20]. Methods for chaotic signal analysis originally arose from a need to rigorously describe physical phenomena that exhibited what was formerly thought to be purely stochastic behavior, but was then discovered to represent complex, aperiodic yet organized behavior, referred to as self-organized dynamics [21]. The analysis of signal complexity on multiple scales may reveal information about neural connectivity that is diagnostically useful [1,19,22].

One interpretation of biological complexity is that it reflects a system's ability to adapt quickly and function in a changing environment [23]. The complexity of EEG signals was found in one study to be associated with the ability to attend to a task and adapt to new cognitive tasks; a significant difference in complexity was found between controls and patients diagnosed with schizophrenia [24]. Patients with schizophrenia were found to have lower complexity than controls in some EEG channels and significantly higher interhemispheric and intrahemispheric cross-mutual information values than controls [25]. A study of the correlation dimension (another measure of signal complexity) of EEG signals in healthy individuals showed an increase with aging, interpreted as an increase in the number of independent synchronous networks in the brain [22].

Several different methods for computing complex or nonlinear time series features have been defined and used successfully to analyze biological signals [26,27]. Sample entropy, a measure of time series complexity, was significantly higher in certain regions of the right hemisphere in preterm neonates who received skin-to-skin contact than in those who did not, indicating faster brain maturation [28]. Sample entropy has also been used as a marker of brain maturation in neonates [29] and was found to increase prenatally until maturation at about 42 weeks, then decreased after newborns reached full term [30].

Living systems exhibit a fundamental propensity to move forward in time. This property also describes physical systems that are far from an equilibrium state. For example, heat moves in only one direction, from hot to cold areas. In thermodynamics, this property is related to the requirement that all systems must move in the direction of higher entropy. Time irreversibility is a common characteristic of living biosignals. It was found to be a characteristic of healthy human heart electrocardiographic (ECG) recordings and was shown to be a reliable way to distinguish between actual ECG recordings and model ECG simulations [31]. ECG signals from patients with congestive heart disease were found to have lower time irreversibility indices than healthy patients [32]. Interestingly, the time irreversibility of EEG signals has been associated with epileptic regions of the brain, and this measure has been proposed as a biomarker for seizure foci [33]. Time irreversibility may be used as a practical test for nonlinearity in a time series.

This study is a preliminary investigation of the difference in multiscale entropy between two groups of infants between 6 and 24 months of age. The groups include typically developing infants and infants who have an older sibling with a confirmed diagnosis of autism spectrum disorder (ASD) and who are thus at higher risk for developing autism. ASD is a developmental disorder in which symptoms emerge during the second year of life. Behavioral indicators are not evident at 6 months of age [34-36]; however, on the basis of the use of a novel observational scale to assess ASD characteristics in infants, distinguishing characteristics were seen at 12 months [35]. Another study compared behavioral measures such as frequency of gaze at faces and shared smiles in infants. Again, group differences between those who later developed an ASD and typically developing controls were apparent at age 12 months, but not at age 6 months [34]. Only one study has investigated behavioral differences at age 9 months: infants at risk for ASD showed distinct differences in visual orientation from those with no family history of autism [37]. These behavioral observations suggest that important developmental differences are occurring in the brains of typically developing infants and those who will later develop an ASD. Although there have been no other published studies on brain development during the first year of life, one of the most replicated findings, based on a retrospective review of medical records, is accelerated growth in head circumference (a valid and reliable proxy for brain growth), which begins at around 6 to 9 months of age [38-40]. If multiscale entropy is a measure of functional brain complexity, then it may be a useful marker for distinguishing differences in brain activity between at-risk and typical infants.

## Methods

### Participants

Data were collected from 79 different infants: 46 who were at high risk for ASD (hereafter referred to as HRA), defined on the basis of having an older sibling with a confirmed diagnosis of ASD, and 33 controls, defined on the basis of a typically developing older sibling and no family history of neurodevelopmental disorders. Testing sessions included infants from ages 6 to 24 months, with some participants tested at more than one age. The study participants were part of an ongoing longitudinal study, and for this analysis visits were evaluated at regular intervals. However, at the time this study was done, most infants had been tested at only one or two visits. Data collected at each session were therefore treated as an independent data set. Thus, the data gathered from an infant who was tested during five different sessions, at ages 6, 9, 12, 18 and 24 months, were treated as unique data sets. Data were collected from a total of 143 sessions and from 79 different individuals. The distribution at different ages and risk groups is shown in Table 1. The number of infants who were tested at only one age at the time of this study is shown in Table 2, as well as the number of infants tested two, three, four and five times. Only one infant thus far has been tested at all five ages from 6 to 24 months. For the purposes of this study, all visits were treated as independent measurements. No comparison of different ages or of growth trajectories between individuals was done. Other characteristics recorded include height and head circumference as shown in Table 1.

**Table 1:** Distribution of participants by age and risk group^a^

	Age									
	
	6 months		9 months		12 months		18 months		24 months	
	
Parameter	HRA	CON	HRA	CON	HRA	CON	HRA	CON	HRA	CON
Number of infants	14	16	16	12	23	17	15	7	14	9
Males, *n *= 59	6	6	8	4	10	6	8	3	4	4
Females, *n *= 84	8	10	8	8	13	11	7	4	10	5
Total, *N *= 143	30		28		40		22		23	
Demographic information										
Mean age, days	189	185	272	273	366	362	549	541	725	727
SD	11.7	8.6	5.1	3.6	9.4	9.0	12.4	6.2	9.1	12.4
Mean height, in	26.5	26.1	27.8	27.2	29.8	29.5	32.1	32.1	34.1	34.8
SD	1.9	1.0	0.7	1.6	1.0	1.5	1.7	1.2	1.1	1.2
*P *value	0.46		0.18		0.53		0.97		0.24	
Mean head circumference, mm	434	435	**459**	**447**	465	466	484	481	492	493
SD	12.7	12.2	**13.7**	**15.8**	12.5	18.0	11.4	18.8	16.7	17.2
*P *value	0.93		**0.04**		0.87		0.61		0.53	
mMSE over channel groups										
Total mMSE	2.02	1.93	2.07	2.02	2.05	1.87	2.16	1.97	2.07	1.96
SD	0.15	0.21	0.20	0.36	0.20	0.35	0.22	0.10	0.14	0.15
*P *value	0.17		0.71		0.07		**0.01**		0.13	
Frontal mMSE	2.02	1.93	2.12	2.08	2.10	1.94	2.18	2.01	2.08	2.00
SD	0.15	0.21	0.20	0.36	0.20	0.35	0.22	0.12	0.11	0.13
*P *value	**0.04**		0.39		0.11		**0.04**		0.21	
Left frontal mMSE	1.94	1.81	1.94	1.91	2.01	1.82	2.06	1.91	2.03	1.88
SD	0.15	0.20	0.20	0.31	0.16	0.32	0.21	0.13	0.13	0.15
*P *value	**0.05**		0.72		**0.04**		0.07		**0.03**	

^a^A total of 79 different infants (46 HRA and 33 CON) participated in this study. Some infants participated in multiple sessions at different ages, raising the total to 143 recording sessions. Also shown are measured demographic variables (age, height and head circumference) and mean multiscale entropy (mMSE) values over three regions: whole head, frontal and left frontal. Statistically significant differences between HRA and CON groups are highlighted in boldface. HRA, high risk for autism, CON, controls; SD, standard deviation.

**Table 2:** Distribution of participants with number of visits and/or measurements of the same child at different agesa

Population	HRA	CON
Number of infants with one time point		
Age 6 months	2	6
Age 9 months	5	2
Age 12 months	4	4
Age 18 months	5	2
Age 24 months	5	3
Total	21	24
Number of infants with two time points	16	8
Number of infants with three time points	8	5
Number of infants with four time points	0	2
Number of infants with five time points	1	1
Total unique infants	46	33
Total measurements, all visits	82	61

^a^Overall, 79 infants participated in the study, and 143 measurement sessions were conducted. HRA, high risk for autism; CON, controls.

The larger Infant Sibling Project study, from which data for this project were taken, was approved by the Committee on Clinical Investigations at Children's Hospital Boston (X06-08-0374) and the Boston University School of Medicine (H-29049). Parental written informed consent was obtained after the experimental procedures had been fully explained.

### EEG data collection

Infants were seated on their mothers' laps in a dimly lit room while a research assistant engaged the infants' attention by blowing bubbles. This procedure was followed to limit the amount of head movement by the infant that would interfere with the recording process. Continuous EEG recordings were taken with a 64-channel Sensor Net System (EGI, Inc., Eugene, OR, USA). This sensor net device comprises an elastic tension structure forming a geodesic tessellation of the head surface and containing carbon fiber electrodes embedded in pedestal sponges. At each vertex is a sensor pedestal housing an Ag/AgCl-coated, carbon-filled plastic electrode and a sponge containing a saline electrolyte solution. Prior to fitting the sensor net over the scalp, the sponges are soaked in electrolyte solution (6 mL of KCl per 1 L of distilled water) to facilitate electrical contact between the scalp and the relevant electrode. To ensure the safety and comfort of the infant, the salinity of the electrolyte solution is the same as tears. In the event that the solution comes into contact with the eyes, no damage or discomfort to the infant will occur.

Prior to recording, measurements of channel gains and zeros were taken to provide an accurate scaling factor for the display of waveform data. The baby's head was measured and marked with a washable wax pencil to ensure accurate placement of the net, which was then placed over the scalp. Scalp impedances were checked online using NetStation (EGI, Inc.), the recording software package that runs this system. EEG data were collected and recorded online using NetAmps Amplifiers (EGI, Inc.) and NetStation software. The data were amplified, band-pass filtered at 0.1 to 100.0 Hz and sampled at a frequency of 250 Hz. They were digitized with a 12-bit National Instruments Board (National Instruments Corp., Woburn, MA, USA). Typically, 2 minutes of baseline activity were recorded, but depending on the willingness of the infant, recorded periods may have been shorter. For this study, continuous sample segments of 20 seconds were selected from the processed resting state data and used to compute multiscale entropy values.

#### Modified Multiscale Sample Entropy

A multiscale method for computing the entropy of biological signals was developed by Costa *et al. *[23]. This approach computes the sample entropy on the original time series (or "signal") and on coarse-scaled series that are derived from the original signal. Because biological systems must be adaptable across multiple time scales, measurements of biological signals are likely to carry information across multiple scales. A multiscale estimation of the information content of EEG signals may reveal more information than the entropy of only the original signal.

Multiple scale time series are produced from the original signal using a coarse-graining procedure. The scale 1 series is the original time series. The scale 2 time series was obtained by averaging two successive values from the original series. Scale 3 was obtained by averaging every three original values and so on as shown in equation (1):

(1) $$\begin{array}{l} s_{1}:x_{1},x_{2},x_{3}\ldots x_{N}\\ s_{2}:\left(x_{1}+x_{2}\right)/2,\left(x_{3}+x_{4}\right)/2,\ldots ,\left(x_{N-1}+x_{N}\right)/2\\ \vdots \\ s_{20}:\left(x_{1}+\cdots +x_{20}\right)/20,\ldots ,\left(x_{N-20}+\cdots +x_{N}\right)/20 \end{array}$$

Coarse-grained series up to scale 20 are computed for each of the 64 EEG channels. The modified sample entropy (mSE) defined by Xie *et al. *[41] was used to compute the entropy of each coarse-grained time series. The mSE algorithm uses a sigmoidal function to compare vector similarity rather than a Heaviside function with a strict cutoff as with the sample entropy used for analysis of biological and ECG signals by Costa *et al. *[23,31]. The practical effect of using the mSE is that the computed entropy values are more robust to noise and the results are more consistent with short time series. In brief, the similarity functions $A_{r}^{m}$ and $B_{r}^{m}$ defined by equations (7) and (9) in the paper by Xie *et al. *[41] are computed with *m *= 2 and *r *= 0.15 for each coarse-grained time series defined in equation (1). The modified multiscale entropy (mMSE) is defined as the series of mSE values at each of the coarse-grained scales from 1 to 20. The mMSE for scale *s *with a finite length time series is then approximated by calculating the following:

(2) mMSE(s,m,r)=−ln⁡(Arm(s)Brm(s)).

**Figure 1:**
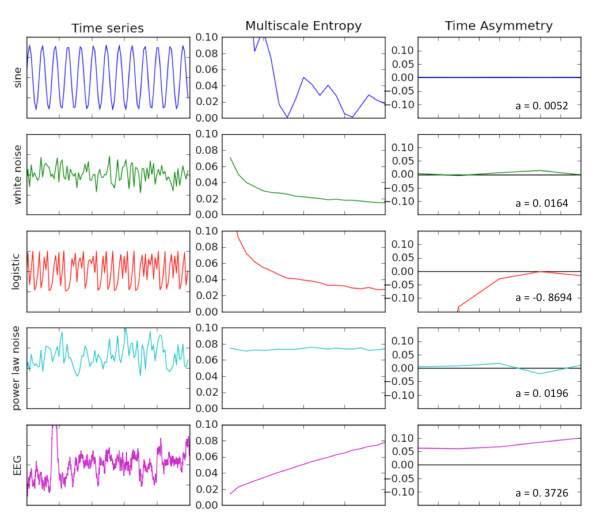
**Characteristics of five different time series are shown**. Column 1 shows the time series amplitudes. Column 2 represents the multiscale entropy, where the horizontal axis is the coarse-grained scale from 1 to 20. Column 3 is the multiscale time asymmetry value. The value of *a *in the lower right corner of the time asymmetry plot is the value of the time asymmetry index summed over scales 1 to 5. A nonzero time asymmetry value is a sufficient condition for nonlinearity of a time series.

The multiscale entropy for several linear, stochastic and nonlinear time series is shown in Figure 1, along with representative mMSE for EEG signals from the EEG data used in this study. The purely random white noise and the completely deterministic logistic equation have similar mMSE curves and visually appear indistinguishable. As discussed by Costa *et al. *[23], these are quite distinct from normal physiological signals. The EEG signal is the only one of the series in Figure 1 that has an mMSE that increases with scale, indicating longer-range correlations in time. Decreasing entropy in general indicates that a signal contains information only on the smallest time scales. If entropy values across all scales for one time series are higher than for another, then the former is considered to be more complex than the latter. Although the mean mMSE value can be computed and used for comparing the overall complexity of physiological signals, the shape of the curve itself may be important for distinguishing two signals.

### Time asymmetry and nonlinearity

The time irreversibility index (*t*_rev_) was computed for different resolutions of the EEG time series using the algorithm of Costa *et al. *[31]. The third column of Figure 1 shows *t*_rev _values for several different linear and nonlinear time series. Of particular note is that only the sine wave time series and both random time series have nearly zero irreversibility indices, while the index for the nonlinear logistic series and the representative EEG signal are both nonzero on all scales shown.

After computing multiple resolutions of the EEG time series as described above, an estimate of the time irreversibility for each resolution was computed by noting that a symmetric function or time series will have the same number of increments as decrements. That is, the number of times |x_*i*+1 _- x_*i*_| > 0 will be approximately the same as the number of times |x_*i*+1 _- x_*i*_| < 0. Thus, an estimate of the time series symmetry (or reversibility) was found by summing increments and decrements and dividing by the length of the series. A reversible time series will have a value of zero. For a series of 5,000 points, as used in this study, *t*_rev _> 0.1 is a significant indicator of irreversibility and thus of nonlinearity [42]. This information is used only to indicate that nonlinear information is contained in the EEG time series that is not used in linear analysis methods, suggesting that the mMSE may contain more diagnostically useful information than power spectra analysis alone.

### Classification and endophenotypes

The Orange machine learning software package (orange.biolab.si/) was used for classification calculations [43]. Several different learning algorithms were compared (support vector machine, *k*-nearest neighbors and naïve Bayesian algorithms) to exclude possible overfitting by one method. The significance of the classification results for each method was estimated empirically using the permutation approach described by Golland and Fischl [44].

To keep the feature set smaller while still capturing the overall shape of the mMSE curve, the low, high and mean values for each curve were extracted for each of 64 channels, creating a feature set of 192 values. A single sample from the population is represented by these 192 values. Although some data points were from the same infant at different ages, this study should be considered a cross-sectional study in that any relationship between data at two different ages was not used for classification. That is, the infants in the age 6 months EEG data set were considered to be independent of the set of infants studied at age 9 months, age 12 months and so on.

## Results

The multiscale entropy and time irreversibility characteristics of five different time series are shown in Figure 1. The example time series amplitudes are shown in the first column. The second column displays plots of the multiscale entropy, where the horizontal axis is the coarse-grained scale from 1 to 20. White noise shows a characteristic decline in entropy with temporal scale, indicating loss of correlation between longer time intervals. Note that the deterministic but chaotic logistic equation has an entropy profile similar to white noise, suggesting that signal characteristics that appear as noise may in fact contain significant dynamic information about the system. The physiological (EEG) time series has a unique entropy curve that increases with temporal scale, similar to the cardiac signals observed in ECG readings [31,45].

The third column of Figure 1 is the multiscale time asymmetry value. The value of *a *in the lower right corner of the time asymmetry plot is the value of the time asymmetry index summed over scales 1 to 5. A nonzero time asymmetry value is a sufficient condition for nonlinearity of a time series. Although white noise and the logistic curve have similar entropy profiles, the time asymmetry index distinguishes the nonlinear chaotic signal from noise. The EEG signal shown here clearly contains nonlinear characteristics on the basis of the nonlinear time asymmetry index.

**Figure 2:**
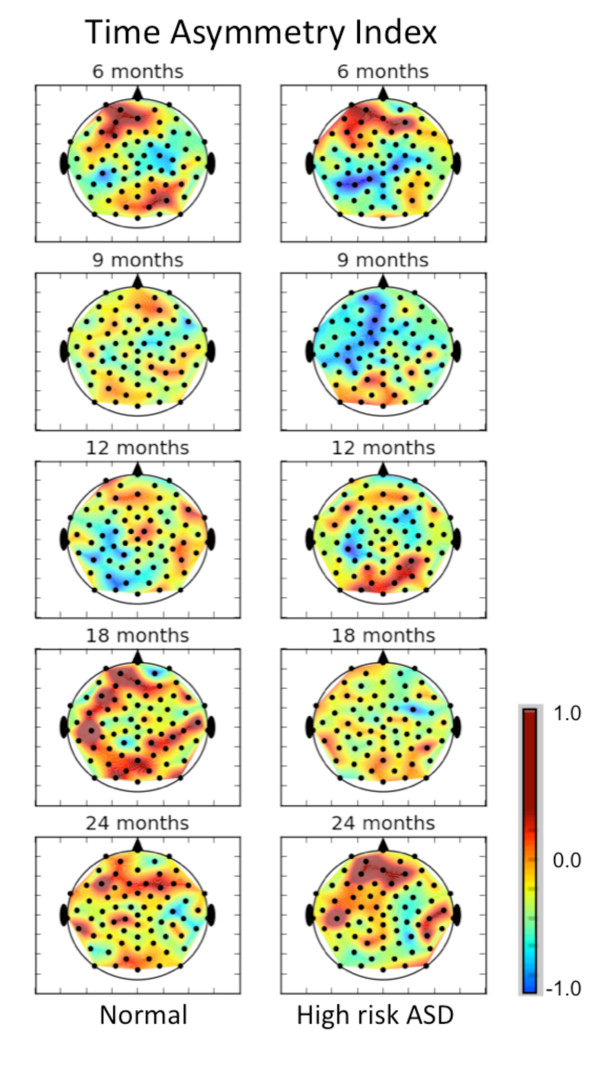
**Time asymmetry index for typical control group and the group of infants at high risk for autism is shown**. The index was averaged over all infants in the group and age categories. If time asymmetry varied randomly at channel locations, the fluctuations would average out. The persistence of time asymmetry values different from zero indicates nonlinearity in the signal.

Using all of the EEG data, we first calculated time asymmetry to determine the degree of nonlinearity present in the signals. Figure 2 shows the time asymmetry index for all 64 channels of the resting state EEG for control and high-risk groups by age. The value of the time asymmetry index in the scalp plot was determined by averaging the index value over all members of that age and risk group. Since the value may take on positive or negative values and will be near zero for time-reversible signal, the persistence of the nonzero values in this plot is an indicator of signal nonlinearity. The multiscale entropy and *t*_rev _values have independent physiological meanings [31]. Since apparent differences exist between controls and the high-risk group at all ages for both mMSE and *t*_rev_, these two quantities together may provide a more sensitive biomarker for developmental age and atypical development. However, in this study, only the multiscale complexity was used to classify the high-risk group.

**Figure 3:**
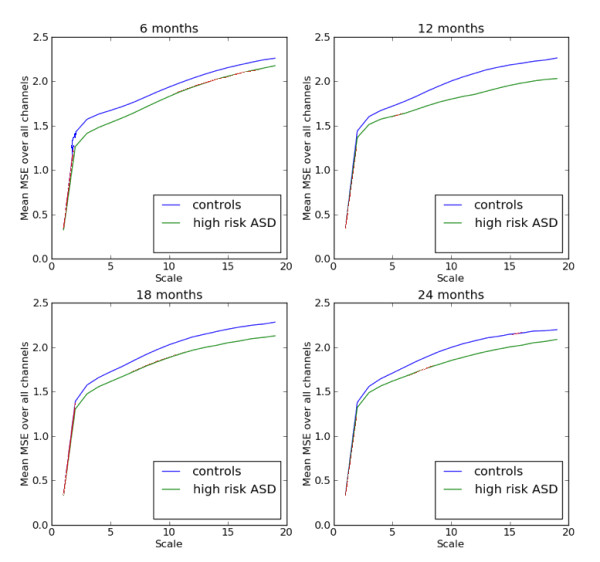
**Modified multiscale entropy (mMSE) is computed for each of 64 channels and for each of the risk groups and averaged over the sample population to produce the mMSE plots for infants ages 6 to 24 months**.

**Figure 4:**
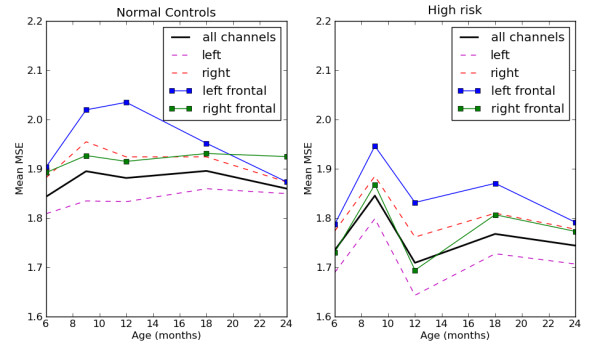
**The change in mean modified multiscale entropy (mMSE) over all channels is shown for each age**. Averaging over all channels reveals that, in general, mMSE is higher in the typical control group than in the group of infants at high risk for autism, but regional differences cannot be seen. Numerical data, including the statistical significance of group differences, are contained in Table 1.

To make some general comparisons of EEG complexity between risk groups and different ages, mMSE curves were averaged over all members of subgroups by both age and risk group. Figure 3 shows that the HRA group had a consistently lower mean complexity over all channels, across all scales and at all ages. Figure 4 shows the group average mMSE value versus age for infants in each of the two risk groups. The bold black line in Figure 4 represents the mean mMSE value averaged over all 64 EEG channels. Left and right laterality were determined by averaging all left-side and all right-side channels separately. Similarly, mMSE values for four left frontal and four right frontal channels were averaged and plotted versus age. Note that the data in Figure 4 are treated as if drawn from a cross-sectional study as described previously. Mean values, standard deviations and statistical significance (*P *values from *t*-test) for the channel averages are given in Table 1. Differences between group averages are significant at age 18 months for overall mean mMSE, and the differences are significant for the left frontal region at all ages except 9 months. Of note is that significant differences were not found at age 9 months for any of the three MSE averages in Table 1, although head circumference was significantly different only at age 9 months. As discussed below, when all mMSE data were considered without averaging (that is, mMSE curves at each channel), machine learning algorithms found the greatest classification accuracy at age 9 months. Although it appears in Figure 4 that the most prominent difference between the control and HRA groups was the change in mMSE between ages 9 and 12 months, significance levels were not computed for changes in this study because measurements at each age were taken from different populations of infants.

Several features are immediately apparent. A general asymmetry in mMSE is seen in both control and high-risk groups, although this asymmetry appears to decline from ages 12 to 18 months as the left and right hemispheres and frontal curves come closer together at age 18 months. EEG complexity changes with age, but not uniformly. In the controls, the overall EEG complexity, shown by the solid black line in Figure 4, increases from ages 6 to 9 months then decreases slightly from ages 9 to 12 months before increasing again from ages 12 to 18 months. Left and right channels and the right frontal channels all follow this same pattern, though left and right hemisphere complexity is not symmetric. The left frontal channels follow a different pattern, increasing strongly until age 12 months and then declining after that. The complexity curves for the high-risk group follow a similar pattern, but the overall complexity is lower and the increases and decreases are much more exaggerated. Perhaps even more distinct is the left frontal curve in the high-risk group. It follows the same pattern as all other regions, unlike the left frontal curve in the controls.

**Figure 5:**
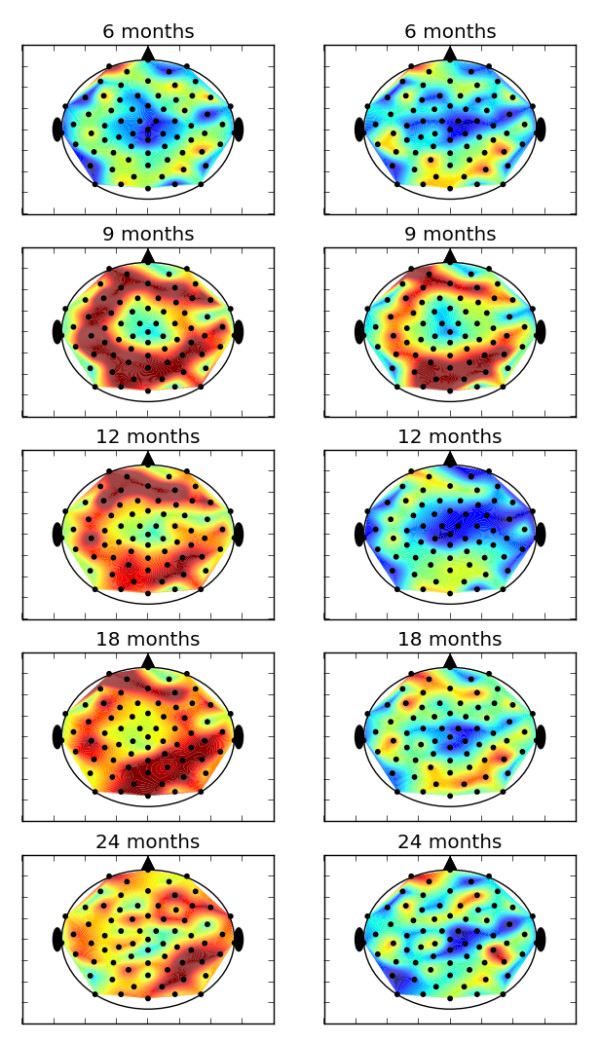
**Mean modified multiscale entropy in each electroencephalography channel averaged over all infants at each age in (a) the typical control group or (b) the group of infants at high risk for autism**.

Since the complexity changes seem to vary with EEG channel, a better picture of complexity development with age and between risk groups can be seen in a scalp plot. Figure 5 shows the mean mMSE value for all EEG channels by risk group and age. The complexity values here were computed by averaging the mMSE over all coarse-grained scales for that channel as in Figure 2. Complexity variation with age and between risk groups is immediately apparent. One or two channels of the left frontal region appear to increase in complexity continuously with age in the controls, as does the right parieto-occipital region. The overall complexity in the high-risk group was lower than in the control group. Although the pattern of complexity change from ages 6 to 9 months appears similar in both groups, the high-risk group shows a marked decline in overall complexity from ages 9 to 12 months.

Height, head circumference and exact age in days at the time of testing, as well as group means, standard deviations and significance levels, are included in Table 1. The only significant group difference among these variables was in head circumference at age 9 months: The infants in the HRA group had a larger mean head circumference than the typically developing controls.

### Machine learning classification of risk

**Figure 6:**
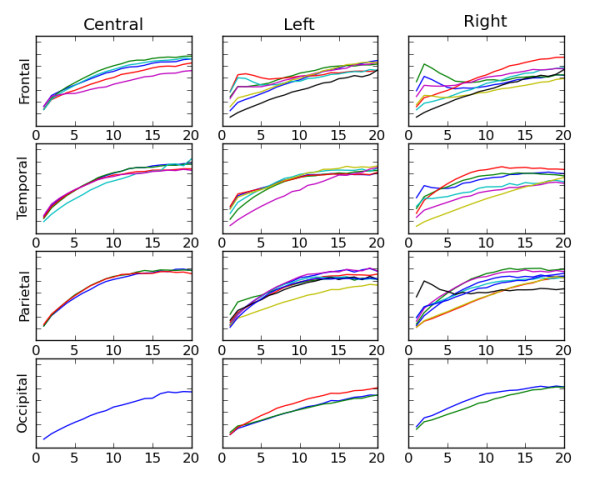
**Mean modified multiscale entropy curves for all 64 channels grouped by brain region for a single 9-month-old infant from the typical control group**. Higher low spatial region (corresponding to high frequency) entropy in the frontal region is one distinct difference in the control example compared to the infants at high risk for autism example in Figure 7.

**Figure 7:**
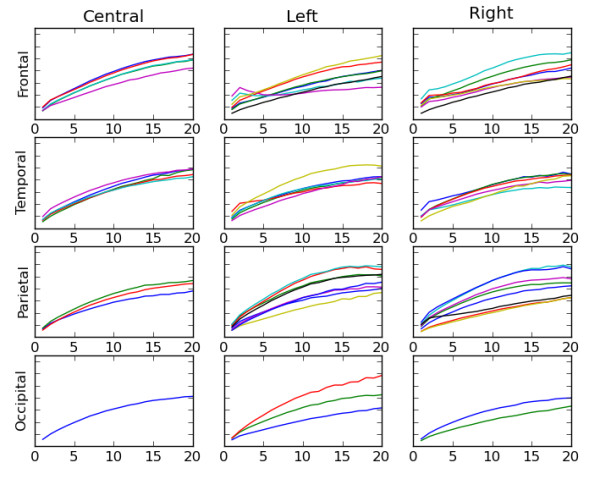
**This figure is analogous to Figure 6, but for a single 9-month-old infant from the high risk group**. Figures 6 and 7 illustrate that the shape of the modified multiscale entropy curve may contain information not seen when using averages alone as in previous scalp plots.

Statistical averages can sometimes obscure meaningful information in complex and highly varying time series. The scalp plots shown in Figure 5 reveal differences between risk groups and ages, but may not use all the information available in the mMSE calculations. For example, the complete mMSE curves on 20 resolutions or scales are shown in Figures 6 and 7 for individual 9-month-old infants. Figure 6 is derived from an infant from the control group, and Figure 7 is derived from an infant from the high-risk group. Curves are grouped by brain region, with 64 curves in all. The purpose of these graphs is simply to illustrate that the shape of the mMSE curves can vary between channels and individuals in distinct ways and that these differences will not be seen in average values. We note that the low spatial scale entropy in the frontal region of the infant from the control group is especially high, while this feature is lacking in the infant from the high-risk group. Although differences between these two examples are apparent, it may be quite difficult to compare 64 mMSE curves for a large number of infants in each group and determine the differences. To use all 64 × 20, or a total of 1,280, multiscale entropy values for each participant, a multiclass support vector machine (SVM) algorithm was used to perform supervised classification of the control and HRA groups.

Using 10-fold cross-validation, infants were classified into either control or high-risk groups using three different learning algorithms as described previously. Since the complexity of all channels is changing rapidly from ages 6 to 24 months, classification within age groups was done rather than comparing the two groups using infants across the entire age spectrum. Machine classification calculations were done for boys and girls together at each age as well as separately. The results of these simulations are shown in Table 3. Classification by age and sex are shown with accuracy and significance estimates for three different machine learning algorithms: the *k*-nearest neighbors (*k*-NN), SVM and naïve Bayesian classification (Bayes) algorithms.

**Table 3:** Supervised learning classification using three different algorithms: *k*-nearest neighbors, support vector machine, and naïve Bayes classification^a^

		Age				
		
Population		6 months	9 months	12 months	18 months	24 months
	*k*-NN	0.67 (0.06)	**0.77** (0.02)	0.53 (0.38)	0.72 (0.12)	0.53 (0.47)
All infants Accuracy (*P *value)	SVM	0.63 (0.16)	**0.77** (0.00)	0.53 (0.71)	0.65 (0.56)	0.55 (0.64)
	Bayes	**0.70** (0.05)	**0.72** (0.03)	0.68 (0.06)	**0.80** (0.04)	0.57 (0.33)
	*k*-NN	0.40 (0.64)	**0.90** (0.00)	0.70 (0.16)	**0.90** (0.03)	-
Boys Accuracy (*P *value)	SVM	0.30 (0.42)	**1.00** (0.00)	0.75 (0.12)	0.75 (0.81)	-
	Bayes	0.35 (0.58)	0.75 (0.10)	0.75 (0.09)	**0.90** (0.05)	-
	*k*-NN	**0.80** (0.03)	0.60 (0.20)	0.48 (0.58)	0.35 (0.88)	0.40 (0.89)
Girls Accuracy (*P *value)	SVM	**0.80** (0.02)	0.40 (0.54)	0.35 (0.97)	0.55 (0.78)	0.75 (0.53)
	Bayes	0.75 (0.07)	0.65 (0.19)	0.47 (0.54)	0.45 (0.73)	0.50 (0.92)

^a^Tenfold cross-validation was run using the computed mean mMSE values on 64 channels for each infant within each age group. *P *values were estimated empirically using a permutation of class labels approach as described in the methods section under 'classification and endophenotypes. Identical cross-validation calculations with 100 permutations were performed to determine empirical *P *values with three different populations: all infants, boys only and girls only. Too few 24-month-old boys were available for cross-validation. *k*-NN, *k*-nearest neighbors algorithm; SVM, support vector machine algorithm; Bayes, naïve Bayes classification algorithm. Boldface entries highlight values with statistical significance of p < 0.05.

The significance of classification accuracy was assessed empirically using the permutation strategy described by Golland and Fischl [44]. This approach is common for estimating the significance of learning algorithms when the number of features greatly exceeds the number of training examples. If the class labels are randomly permutated, new classification accuracy can be computed using 10-fold cross-validation to serve as a baseline. For this study, 100 random permutations were run with 10-fold cross-validation for each machine classification calculation. The *P *value was determined by counting the number of random classifications for which the accuracy was equal to or higher than the accuracy for the true labels.

Using *P *= 0.05 as a significance cutoff value, the HRA and control groups can be classified at age 9 months for boys and girls together and for boys separately with accuracies of nearly 80% and well over 90%, respectively. For boys considered alone, the classification accuracy remained relatively high at ages 9, 12 and 18 months, though the result at age 12 months was not statistically significant. For girls, separation of the two groups was most accurate and significant at age 6 months, possibly indicating a sex difference in developmental trajectories. These results suggest that a familial endophenotype may be present at around age 9 months that enables HRA infants to be distinguished from low-risk controls. The differences seem to decline after 9 months of age, especially in girls, with some evidence that it may persist in boys until age 18 months (Table 3). Since approximately 60% of the HRA infants are expected not to be diagnosed with an ASD (20% will likely be diagnosed with another disorder, although not an ASD) [36], this is not surprising. Increasing heterogeneity with age regarding rates of development and behavioral characteristics of the high-risk group may be partly responsible for the drop in accuracy. Further study and subclassification with future data are needed to explore sex differences in brain development using entropy calculations.

To determine whether the significant group differences in mean head circumference were predictors of individual class status, two additional calculations were done. First, head circumference was added as one more feature to the mMSE values. The prediction calculations were repeated. The predictive accuracy of the classifiers was unchanged from the results obtained with mMSE alone. This might have been because the changed mMSE values were a direct reflection of head size differences in some way, so classification was done with head circumference alone. Somewhat surprisingly, classification accuracy was not significant and nearly random. When examining the group values, it appears that the rather large individual variability within each group accounts for this finding. We conclude that head circumference does not contribute to classification accuracy at any of the ages tested.

## Discussion

The primary goal of this study was to explore whether measures of EEG complexity might reveal functional endophenotypes of ASD and thus identify them as potential biomarkers for risk of ASD at very early ages before the onset of clear behavioral symptoms. Our findings show significant promise for the specific measure of multiscale entropy that was used to compare high- and low-risk infants between the ages of 6 and 24 months. Differences in mean mMSE over the entire scalp and especially in the left frontal region were significant at most ages measured, except at age 9 months. The trajectory of the curves between ages 6 and 12 months in Figure 4 appears to be as informative as information at any specific age. This result makes the relatively high accuracy at age 9 months of the machine classification using all of the mMSE curves as feature vectors particularly notable. This early period of life is one of important changes in brain function that are foundational for the emergence of higher-level social and communicative skills that are at the heart of the difficulties associated with ASD. A number of major cognitive milestones typically occur beginning at around age 9 months and perhaps earlier in girls. These milestones include, for example, the development of the ability to perceive intentional actions by others [46], as well as loss of the ability to perceive speech sound distinctions in non-native languages [47] and loss of the ability to discriminate certain categories of faces [48]. These latter developments are especially significant because they reveal how socially grounded experiences influence changes in the neurocognitive mechanisms that underlie speech and face recognition processing. Thus, Marcus and Nelson [49] argued that infants mold their face-processing system on the basis of the visual experiences they encounter, just as their speech-processing skills are molded to their native language [50,51]. This model assumes a narrowing of the social-perceptual window through which language and faces are processed, which in turn results in an increase in cortical specialization. In a prospective study, Ozonoff *et al. *[34] found that social communicative behaviors in infants who later developed ASD declined dramatically between ages 6 and 18 months compared to typically developing infants.

We hypothesize that the following developmental sequence may explain the data in Table 3. At age 6 months, no significant behavioral differences have been noted in prospective studies between typically developing infants and those who develop autism [34,35]. Thus, few differences in electrophysiological data are expected at age 6 months, as shown in Figure 4 and Table 3. However, if girls are considered separately, differences in mMSE appear to be significant at age 6 months. If the multiscale entropy calculations from the EEG signals are indeed a biomarker for endophenotypes of autism familial traits, then by 9 months of age many infants in the high-risk group will display unique characteristics in their mMSE profiles that enable them to be distinguished from the controls. Those infants in the high-risk group who do not have multiple risk factors and later develop normally would not be expected to exhibit abnormalities in their mMSE profiles throughout the developmental period. These hypotheses might account for the HRA infants in our study who were classified similarly to our typical controls. This hypothesis will be tested when sufficient numbers of infants in the HRA group have reached 2 to 3 years of age and a diagnosis of ASD or typical development can be made.

Developmental abnormalities from ages 6 to 12 months are particularly distinct in the two groups (low and high risk for ASD), allowing the groups to be classified quite accurately, although some overlap between the HRA and control groups should be expected at all ages. From 12 to 24 months of age, the distinction between the two groups declines. This likely reflects the trend for some fraction of high-risk infants to develop more typical cognitive and behavioral function, even though they may carry endophenotypes that share common complexity profiles at an earlier age with other high-risk infants who will later be diagnosed with ASD.

Rather than analyzing entropy at single age points, using a trajectory of entropy values from ages 6 to 24 months might be more informative. Although EEG complexity has been shown in several studies to increase with age [30,52,53], the increase is neither monotonic nor uniform across different brain regions. The abnormalities in brain development that lead to autistic characteristics may not be immediately apparent by inspecting relevant brain activity, even if the data contain diagnostically significant information. For example, a recent study of the relationship between cortical thickness and intelligence found no correlation between absolute cortical thickness at any particular age and intelligence. However, a specific pattern of developmental changes in cortical thickness was highly correlated with intelligence [54].

One of the characteristics of the high-risk group is heterogeneity: This group includes infants who will go on to develop an ASD and those who are within the normal range genetically, developmentally and behaviorally, as well as those in between who exhibit mild autism-like traits. Further study of this cohort as they grow and develop will enable this hypothesis to be tested. Rather than binary classification into typical controls and heterogeneous high-risk groups, classification on the basis of actual behavioral assessments will allow a more accurate test of the efficacy of using the mMSE to measure brain function.

## Conclusions

Abnormal brain connectivity, whether locally, regionally or both, may be a cause of a number of behavioral disorders, including ASD [9], and changes in local complexity are believed to be related to brain connectivity [55]. Local neural network connectivity undergoes rapid change during early development, and this may be reflected in the multiscale entropy of EEG signals, which is one measure of signal complexity that has been associated with health and disease [23]. A number of recent studies have demonstrated a link between brain connectivity and complexity, and EEG signal complexity may provide valuable information about the neural correlates of cognitive processes [56]. Early markers for neurological or mental disorders, particularly those with developmental etiologies, may be the growth trajectories of complexity as measured by multiscale entropy curves. The results described in this paper suggest that infants in families with a history of ASD have quite different EEG complexity patterns from 6 to 24 months of age that may be indicators of a functional endophenotype associated with ASD risk. Differences between mean mMSE averaged over all channels or in frontal regions in the two groups are significant at all ages except 9 months. Machine classification on the basis of mMSE curves in each channel as a feature set is able to determine group membership, particularly at 9 months of age. The classification accuracy decreases after age 12 months, possibly because of the influence of normal brain development and the development of normal characteristics in many of the high-risk infants. Classification accuracy for boys alone still appears to be significant and relatively high at age 18 months. More data about the future outcomes of the HRA infants and the computation of additional features, such as laterality of entropy, together with behavioral and cognitive assessments as the cohort of participants in this study grows, may enable the high-risk population to be subclassified more accurately. Future longitudinal analysis of data from this cohort will allow growth trajectories, as well as the future outcomes of the high-risk children, to be compared. Deeper understanding of the relationship between these neurophysiological processes and cognitive function may yield a new window to the mind and provide a clinically useful psychiatric biomarker using complexity analysis of EEG data.

## Competing interests

WJB is named on a provisional patent application submitted by the Children's Hospital Boston Technology Development Office that includes parts of the signal analysis methods discussed in this article. The authors declare that they have no other competing financial or nonfinancial interests.

## Authors' contributions

WJB conceived of the analytical methods used in this paper, wrote needed computer codes, performed calculations and statistical analysis and drafted the manuscript. AT carried out the initial processing of the raw data, participated in discussion of analysis results and contributed to drafting the methods section. CAN and HTF are co-Principal Investigators on the larger Infant Siblings Project study upon which this paper was based, contributed to the study design, interpretation of developmental implications of the results and were responsible for coordinating recruitment and testing of all patient data. All authors read and approved the final manuscript.

## Pre-publication history

The pre-publication history for this paper can be accessed here:

http://www.biomedcentral.com/1741-7015/9/18/prepub
